# Identifying specific proteins involved in eggshell membrane formation using gene expression analysis and bioinformatics

**DOI:** 10.1186/s12864-015-2013-3

**Published:** 2015-10-15

**Authors:** Jingwen Du, Maxwell T. Hincke, Megan Rose-Martel, Christelle Hennequet-Antier, Aurelien Brionne, Larry A. Cogburn, Yves Nys, Joel Gautron

**Affiliations:** Department of Cellular and Molecular Medicine, University of Ottawa, 451 Smyth Road, Ottawa, K1H 8 M5, Canada; INRA, UR83 Recherches Avicoles, F-37380, Nouzilly, France; Department of Animal and Food Sciences, University of Delaware, Newark, DE 19717 USA

**Keywords:** Chicken eggshell membrane, White isthmus, Collagens, Bioinformatics, Transcriptome

## Abstract

**Background:**

The avian eggshell membranes surround the egg white and provide a structural foundation for calcification of the eggshell which is essential for avian reproduction; moreover, it is also a natural biomaterial with many potential industrial and biomedical applications. Due to the insoluble and stable nature of the eggshell membrane fibres, their formation and protein constituents remain poorly characterized. The purpose of this study was to identify genes encoding eggshell membrane proteins, particularly those responsible for its structural features, by analyzing the transcriptome of the white isthmus segment of the oviduct, which is the specialized region responsible for the fabrication of the membrane fibres.

**Results:**

The Del-Mar 14 K chicken microarray was used to investigate up-regulated expression of transcripts in the white isthmus (WI) compared with the adjacent magnum (Ma) and uterine (Ut) segments of the hen oviduct. Analysis revealed 135 clones hybridizing to over-expressed transcripts (WI/Ma + WI/Ut), and corresponding to 107 NCBI annotated non-redundant *Gallus gallus* gene IDs. This combined analysis revealed that the structural proteins highly over-expressed in the white isthmus include collagen X (*COL10A1*), fibrillin-1 (*FBN1*) and cysteine rich eggshell membrane protein (*CREMP*). These results validate previous proteomics studies which have identified collagen X (α-1) and CREMP in soluble eggshell extracts. Genes encoding collagen-processing enzymes such as lysyl oxidase homologs 1, 2 and 3 (*LOXL1, LOXL2 and LOXL3),* prolyl 4 hydroxylase subunit α-2 and beta polypeptide (*P4HA2* and *P4HB)* as well as peptidyl-prolyl cis-trans isomerase C (*PPIC*) were also over-expressed. Additionally, genes encoding proteins known to regulate disulfide cross-linking, including sulfhydryl oxidase (*QSOX1*) and thioredoxin (*TXN*), were identified which suggests that coordinated up-regulation of genes in the white isthmus is associated with eggshell membrane fibre formation.

**Conclusions:**

The present study has identified genes associated with the processing of collagen, other structural proteins, and disulfide-mediated cross-linking during eggshell membrane formation in the white isthmus. Identification of these genes will provide new insight into eggshell membrane structure and mechanisms of formation that will assist in the development of selection strategies to improve eggshell quality and food safety of the table egg.

**Electronic supplementary material:**

The online version of this article (doi:10.1186/s12864-015-2013-3) contains supplementary material, which is available to authorized users.

## Background

The cleidoic avian egg is a highly ordered structure and the recognized hallmark of reproduction in birds. During egg formation, the accumulation of liver-secreted egg yolk proteins takes place in the chicken ovary. Following ovulation, the developing egg transits through specialized regions of the oviduct where the egg white, eggshell membranes and eggshell are sequentially deposited in the magnum, white isthmus and uterine segments, respectively [1]. The innermost layer of the shell is composed of eggshell membranes that are deposited during the brief (~1 h) passage through the white isthmus [2, 3]. These fibres are organized into inner and outer membranes, which are joined by inter-connecting fibres that form a highly cross-linked fibrous meshwork, arranged in alternating layers parallel to the eggshell surface [4].

Calcification of the eggshell originates at nucleation sites located on the surface of the outer eggshell membranes. These sites develop into the calcified mammillary cones that form the inner layer of the mineralized eggshell. The nature of the template and molecules involved in the initiation of mineral deposition at this array of nucleation sites has recently been explored and revealed the presence of amorphous calcium carbonate as an intermediary phase [5] as well as the presence of numerous proteins [6, 7], the roles of which remain poorly understood. However, this initial phase of calcification is a key step in eggshell biomineralization. The texture of the shell and details of its ultrastructure (i.e. mammillary cone spacing and attachment to the membrane fibres) are influenced by this early stage and are critical for shell strength [8–11]. Animal studies have demonstrated that disruption of eggshell membrane fibres (by alterations in membrane fibre cross-linking and organization) severely reduces eggshell quality and strength [12–14]. These observations reinforce the concept that the membranes are essential elements in the fabrication of a durable eggshell, which resists bacterial contamination in the table egg [15].

Viewed from another perspective, the eggshell membranes have potential as a useful biomaterial and are available in large quantities as a waste product from the egg processing (breaker) industry. They possess unique properties, with industrial, nutraceutical, cosmetic, and biomedical applications [16]. Biomaterials have increasing applications in many useful fields; in order to maximally exploit their function it is essential to fully understand their components and individual properties. Therefore, to optimally exploit the eggshell membranes as a biomaterial, it is necessary to fully characterize its constituents.

Due to the stable, insoluble and highly cross-linked nature of the membrane fibres, there are many unanswered questions about their composition and functional significance of their constituents. Although previous studies defined some proteins that are located in the eggshell membrane fibres [17–19], a complete understanding of all proteins involved in the formation of the eggshell membrane and their role(s) is not yet available. Since the entire oviduct originates from the same population of cells [20], we hypothesize that over-expressed genes in each specialized region of the oviduct could encode proteins that are involved in the formation of a particular egg compartment. With this strategy, previous studies of the hen uterus transcriptome during formation of the eggshell identified a large number of functional genes that participate in eggshell formation and ion transport for its mineralization [21, 22]. Additionally, a recent uterine transcriptomic study which compared the expression of uterine genes in the presence and absence of calcification further updated our knowledge of genes encoding proteins that supply ions for shell formation [23]. In the current study, we report a re-evaluation of the dataset utilized for uterine-specific expression, in order to gain insight into white isthmus-specific functions. We test the hypothesis that comparison of gene expression in the white isthmus, where the eggshell membranes are formed, with two adjacent segments (magnum - where the egg white is formed, and uterus - site of eggshell calcification) will reveal specific over-expressed genes encoding proteins involved in the formation and structure of the eggshell membranes. We have used bioinformatics analysis, such as functional annotation, and gene ontology enrichment to further analyze the proteins encoded by over-expressed white isthmus genes, with confirmation by comparison with the eggshell membrane proteome [17–19].

## Results

### Up-regulated expression of white isthmus genes

The Del-Mar 14 K Chicken Integrated Systems microarray (NCBI GEO Platform # GPL1731) [24] was used for the analysis of over-expressed genes in three segments of the hen oviduct. Our original reports described uterine-specific gene expression relative to other oviduct parts [21], and the validation by qRT-PCR analysis of 16 differentially expressed transcripts detected by microarray analysis of the hen’s oviduct. In the present analysis, we used comparisons of gene expression in the white isthmus (WI) with two other segments (magnum (Ma) or uterus (Ut)) of the hen’s oviduct to predict genes encoding proteins involved in formation of the eggshell membranes.

**Fig. 1:**
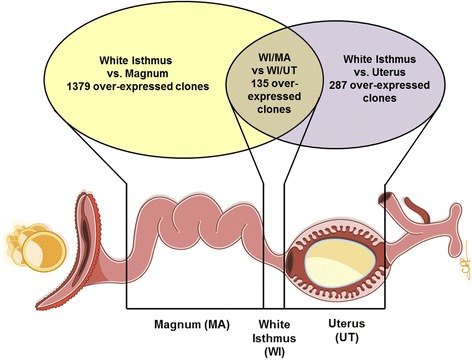
Comparison of clones hybridizing to over-expressed transcripts in WI/Ma and WI/Ut comparisons. Depiction of over-expressed transcripts along the length of the oviduct from magnum (Ma) to white isthmus (WI) to uterus (Ut), as per analysis of individual clones in the microarray. The 135 upregulated transcripts correspond to 107 unique *Gallus gallus* genes (Additional file 1). This image is modified, with permission, from Hincke et al*.,* 2012 [10]

A general assessment was performed to identify all up-regulated transcripts. A total of 1514 transcripts were over-expressed in the white isthmus compared to the magnum while 422 transcripts were over-expressed in the white isthmus compared to the uterus. However, only 135 clones hybridizing to over-expressed transcripts were common to the two comparisons (Fig. 1); according to our hypothesis, these over-expressed genes are related to white isthmus-specific functions (Additional file 1).

The 135 clones that were common to WI/Ma and WI/Ut comparisons were further assessed. Nine of them could not be further analyzed, since either the clone ID was no longer in the current nucleotide GenBank or in the University of Delaware chick EST database, or Blast searching did not reveal any hit. After removing examples of redundant clones, the over-expressed transcripts corresponding to 107 unique Gene IDs were retained and correspond to over-expressed genes in the white isthmus that possess an NCBI non-redundant *Gallus gallus* gene ID.

Functional annotation clustering analysis using DAVID Bioinformatics Resources revealed 2 significantly enriched Gene Ontology (GO) term clusters involved in protein translation and RNA binding/splicing (Table 1). GO term analysis also revealed several genes with possible roles in membrane fibre formation and/or the initiation of calcification (Table 2). Nineteen proteins were categorized as “ion binding”, including carbonic anhydrase 5B (*CA5B*). Of these 19 genes, 7 were also categorized as calcium ion binding, most notably fibrillin-1 (*FBN1*), reticulocalbin 2 (*RCN2*) and calcineurin-like EF-hand protein 1 (*CHP1*). GO term analysis also revealed 3 genes involved in the formation of proteinaceous extracellular matrices, including *FBN1* and collagen X (α-1) (*COL10A1*). Oxidation-reduction reactions are necessary for disulfide mediated cross-linking of eggshell membrane fibres. Among the 4 genes categorized as “oxidation reduction” by GO term analysis are thioredoxin (*TXN*) and prolyl 4-hydroxylase, α polypeptide II (*P4HA2*); the latter is a known collagen-modifying enzyme (Table 2).

**Table 1:** Functional annotation clustering analysis for the 107 genes associated with the eggshell membranes

	GO Terms	*P*-value^a^	Identified genes^b^
Two Identified Clusters			
*Cluster 1*	GO:0006412 ~ translation	3.39E-07	*EEF1B2, RPL31, RPL21, RPS23, RPS24, EIF4A2, EIF3D, MARS2, EPRS, RPL3, EEF1D, RPL11*
E-score^c^ 2.23			
	GO:0003735 ~ structural constituent of ribosome	1.57E-03	*RPL31, RPL21, RPS23, RPS24, RPL3, RPL11*
	GO:0005198 ~ structural molecule activity	5.33E-02	*FBN1, RPL31, RPL21, RPS23, RPS24, RPL3, RPL11*
*Cluster 2*	GO:0003723 ~ RNA binding	1.11E-02	*PUM2, EIF4A2, MBNL1, PABPC1, HDLBP, CUGBP2, YBX1*
E-score 1.14			
	GO:0008380 ~ RNA splicing	5.18E-02	*MBNL1, CUGBP2, YBX1*
Enriched GO Term			
	GO:0008135 ~ translation factor activity, nucleic acid binding	7.35E-03	*EEF1B2, EIF4A2, EIF3D, EEF1D*

^a^ *P*-value corresponds to the EASE score determined by the DAVID software

^b^Gene IDs corresponding to transcripts associated with the eggshell membranes (Additional file 1)

^c^E-score: enrichment score determined by the DAVID software

**Table 2:** GO terms associated with eggshell membrane formation

	GO Terms	Identified Genes^a^
Biological Process		
	GO:0055114 ~ oxidation reduction	*ALDH3A2, P4HA2, SORD, TXN*
Molecular Function		
	GO:0043167 ~ ion binding	*EDEM1, SQSTM1, GCH1, ENO4, P4HA2, ANXA5, MBNL1, CHP1, CA5B, SLC39A13, LIMCH1, FBN1, ADAMTS1, NME2, TPD52, RCN2, NCS1, SORD, PDLIM1*
	GO:0005509 ~ calcium ion binding	*FBN1, EDEM1, ANXA5, CHP1, TPD52, CN2, NCS1*
Cellular Component		
	GO:0031012 ~ extracellular matrix	*FBN1, ADAMTS1, TGFB3, COL10A1*
	GO:0005578 ~ proteinaceous extracellular matrix	*FBN1, ADAMTS1, COL10A1*

^a^Gene IDs corresponding to transcripts associated with the eggshell membranes (Additional file 1)

In Table 3 we list the most highly over-expressed WI genes, which are arbitrarily defined as over-expression ≥ 2-fold (Log2 Ratio ≥ 1.0) relative to either the magnum or the uterus. A relatively small number of genes were in this category (14/107). Two of these genes (*COL10A1* and IgGFc-binding protein-like (*FCGBP*)) were identified by more than one clone, which provides an internal control to validate the reproducible nature of the over-expression assessment. Transcript abundance in the biological samples used for this microarray study was already assessed by quantitative real time PCR (qRT-PCR), as we previously reported [21].

**Table 3:** Highly over-expressed genes identified in the white isthmus during deposition of the eggshell membranes^a^

Gene symbol	Entrez gene name	WI/Ut (fold change)	WI/Ma (fold change)	Secretion		Confirmed by proteomics	
				SignalP^b^	SecretomeP^c^	ES^d^ membranes	ES^d^ matrix
*COL10A1*	Collagen α-1(X) chain-like	111.43; 50.21	155.42; 94.35	Yes	-	[7, 17, 18]	[6, 35, 38]
*R3HCC1*	R3H domain and coiled-coil containing 1	79.34	136.24	-	-	-	-
*CREMP*	PREDICTED: similar to spore coat protein SP75, partial	70.03	58.08	-	Yes	[7, 17, 18]	-
*AP1G1*	Adaptor-related protein complex 1, gamma 1 subunit	45.89	15.56	-	-	-	-
*FBN1*	Fibrillin 1	45.89	2.53	-	-	-	-
*FCGBP*	IgGFc-binding protein-like	22.63; 7.62	34.06; 8.82	Yes	-	-	-
*MXI1*	MAX interactor 1	14.12	9.85	-	-	-	-
*LIMCH1*	LIM and calponin homology domains-containing protein 1	11.63	15.45	-	-	-	-
*FAM164C*	Family with sequence similarity 164, member C	3.71	2.79	-	Yes	-	-
*TDRD9*	putative ATP-dependent RNA helicase	3.36	2.68	-	Yes	-	-
*HPX*	Hemopexin	2.95	1.57	-	Yes	[7, 17, 18]	[6, 35, 38]
*TMEM63C*	Transmembrane protein 63C	2.58	2.16	-	-	-	-
*SORD*	Sorbitol dehydrogenase	2.48	2.25	-	Yes	-	-
*PHGDH*	D-3-phosphoglycerate dehydrogenase	1.79	2.39	-	Yes	-	-
*TXN*	Thioredoxin	1.66	2.06	-	-	[7, 17]	[35]

^a^High over-expression defined as ≥ 2-fold (Log2 Ratio ≥ 1.0) relative to either the magnum or the uterus

^b^Predicted by SignalP 4.1 software

^c^Predicted by SecretomeP 2.0 software

^d^ES Eggshell

*COL10A1* is the only collagen highly over-expressed in the WI. Collagen I (α-1) (*COL1A1*), collagen I (α-2) (*COL1A2*) and collagen III (α -1) (*COL3A1*) were only over-expressed in the WI when compared to the Ma (Table 4). A closer look at collagen-processing enzymes revealed that lysyl oxidase homolog 3 (*LOXL3*) was over-expressed (1.27-fold) in the WI/Ma contrast and lysyl oxidase homolog 2 (*LOXL2*) was over-expressed (18.64-fold) in the WI/Ut contrast (Table 4). A detailed analysis of the expression levels of known collagen and collagen-modifying genes in the chicken oviduct is listed in Table 4.

**Table 4:** Over-expression of collagens and collagen-modifying genes in the chicken oviduct

Gene	Collagen type	Chain	WI/Ut (fold change)^a^	WI/Ma (fold change)^a^	Confirmed by Proteomics	
					ES^b^ membranes	ES^b^ matrix
*COL1A1*	I	α-1	-	1.23	-	[34]
*COL1A2*		α-2	-	1.18	[17]	[34, 38]
*COL2A1*	II	α-1	-	-	-	[6, 35, 38]
*COL3A1*	III	α-1	-	1.43; 1.16	-	[34]
*COL4A1*	IV	α-1	-	-	-	-
*COL4A2*		α-2	-	-	-	-
*COL4A3*		α-3	-	-	[17]	-
*COL5A1*	V	α-1	-	-	-	-
*COL5A2*		α-2	-	-	[17]	[35]
*COL6A1*	VI	α-1	-	-	-	[38]
*COL6A2*		α-2	-	-	[18]	[6, 35, 38]
*COL7A1*	VII	α-1	-	-	-	[38]
*COL8A1*	VIII	α-1	-	-	-	-
*COL10A1*	X	α-1	111.43; 50.21	155.42; 94.35	[7, 17, 31]	[6, 35, 38]
*COL17A1*	XVII	α-1	-	-	-	[6, 38]
*COL18A1*	XVIII	α-1	-	-	-	[35]
*COL20A1*	XX	α-1	-	-	-	-
*COL22A1*	XXII	α-1	-	-	-	-
*COL24A1*	XXIV	α-1	-	-	[19]	-
	Collagen-modifying enzymes					
*LOX*	Lysyl Oxidase	-	-	-	-	-
*LOXL1*	Lysyl oxidase-like 1	-	**	**	-	-
*LOXL2*	Lysyl oxidase-like 2	-	18.64	-	[7, 17]	[6, 33, 38]
*LOXL3*	Lysyl oxidase-like 3	-	-	1.27	[17, 18, 31]	[38]
*LOXL4*	Lysyl oxidase-like 4	-	-	-	[19]	-
*P4HA2*	Prolyl 4-hydroxylase	α-2	1.21	1.64	-	-
*P4HB*	Prolyl 4-hydroxylase	β	-	1.27	-	[6]
*PPIC*	Peptidyl-prolyl cis-trans isomerase C	-	1.28	1.51	[17]	[6, 35, 38]

^a.^ **Expressed at very high (saturating) levels in all three tissues

^b.^ ES - Eggshell

The eggshell membrane fibres are extracellular structures; electron microscopy reveals that they are fabricated in the white isthmus tubular glands and then secreted into the lumen of the oviduct where assembly into the mesh-like membrane structure occurs [25]. The apertures of the tubular glands are more numerous in the white isthmus than in any other oviduct region [26]. Secreted proteins and/or enzymes are candidates to participate structurally or enzymatically in their assembly. The SignalP 4.1 and SecretomeP 2.0 servers were used to interrogate the protein sequences of highly over-expressed genes (Table 3) to search for predictions of protein secretion by classical signal peptide – mediated mechanisms or the unconventional pathway which mediates leaderless secretory proteins. Those with predicted signal peptides included *COL10A1* and *FCGBP*. Similar to spore coat protein SP75, also known as cysteine rich eggshell membrane protein (*CREMP*), is a potential structural protein that possessed sequence determinants for unconventional secretion. However, it is unclear whether the 5’ sequence of this cDNA (coding for the N-terminus) has been identified since its gene has not yet been cloned. Among the 14 genes that were highly over-expressed, only three have previously been confirmed by proteomics studies. *COL10A1*, *CREMP* and hemopexin (*HPX*) have previously been extracted from eggshell membranes derived from both fertilized and unfertilized eggs [6, 7, 17, 18].

## Discussion

The calcareous egg of birds and reptiles, and formerly dinosaurs, has been an extremely successful reproductive adaptation to the desiccating terrestrial environment [10]. The avian eggshell has been shaped through evolution to resist physical and pathogen challenges from the external environment, while regulating gas and water exchange, and serving as a calcium store, to satisfy the metabolic and nutritional needs of the developing embryo. The precursors of the inner and outer eggshell membranes are synthesized in the tubular gland of the white isthmus and then secreted and assembled while the forming egg remains within the white isthmus for approximately 1 h. This meshwork of interlaced fibres is organized into morphologically distinct inner and outer sheets that enclose the egg albumen. The inner membranes remain uncalcified, while the fibres of the outer shell membrane become partly mineralized on the external surface at discrete sites which are points of attachment of the mammillary cones of the inner surface of the calcified shell. The putative keratan sulfate proteoglycan (“mammillan”) associated with the nucleation sites on the external membrane surface has not yet been identified [27–29]. Many ultrastructural features of the calcified eggshell, such as mammillary cone spacing and type of attachment to the underlying membrane fibres are important for shell strength, eggshell quality and ultimately prevention of pathogen contamination of the nutritious table egg. Animal studies have demonstrated that disruption of eggshell membrane fibres (cross-linking and/or organization) or reduced proteoglycan sulfation severely reduces eggshell quality and strength [12–15, 29]. However, the extremely cross-linked nature and insolubility of the membrane fibres has prevented successful approaches to identifying all protein components.

Here, we report a transcriptomic approach to determine the genes/proteins associated with formation and fabrication of eggshell membrane fibres, with the hypothesis that over-expressed genes in the white isthmus, compared to adjacent oviduct segments, are involved in white isthmus-specific functions. Our previous transcriptome study of the chicken uterus during eggshell calcification provided a detailed description of the over-expressed genes that are important for shell formation [21, 23]. This approach was further refined to identify the genes encoding proteins responsible for the ion transport necessary for eggshell mineralization [22, 23]. In the present study, we provide a transcriptional analysis of the white isthmus to gain insight into the genes associated with eggshell membrane formation. We identified 135 cDNA clones hybridizing to over-expressed white isthmus transcripts, corresponding to 107 unique non-redundant genes.

### Collagens and related proteins

The eggshell membrane fibres are not exclusively made of collagens as clearly shown by the amino-acid profile of the eggshell membranes, although they contain at least 10 % collagens (types I, V and X) [1, 15, 18, 27–31]. In our study, the microarray contains two independent clones that are specific for different regions of *COL10A1* and over-expression levels in the white isthmus were extremely high when compared to both the magnum and the uterus (Table 3). Collagen X is a short chain collagen molecule existing as a homotrimer of the α-1 (X) chains, which contributes to structural integrity. *In situ* hybridization and immunohistochemistry have previously revealed that collagen X is mainly expressed in the tubular gland cells of the white isthmus segment of the oviduct [32]. Proteomics studies have detected peptides from collagen X in eggshell membrane extracts from both fertilized and unfertilized eggs [7, 17, 18], within the shell matrix corresponding to the mammillary cones [6, 7], and in the uterine fluid during the initial stage of mineralization [33].

Type I and V collagens are known to be less abundant constituents of the eggshell membranes and represent 4 mg/g of whole eggshell membrane, or about 0.6 % of the membrane protein [30, 31]. In the present study, over-expression of *COL1A1*, *COL1A2* and *COL3A1* were detected in the WI/Ma contrast only (Table 4). No over-expression of collagens other than *COL10A1* was detected in the WI/Ut contrast, suggesting that these genes were expressed at equivalent levels in both white isthmus and uterine segments of the oviduct. This observation is supported by recent proteomics analysis where collagen X (α-1) and collagen II (α-1) were detected in eggshells collected at the early stage of calcification [6]. Proteomics analyses of chicken eggshell membranes have also confirmed the presence of collagens type I (α-2), IV (α-3), V (α-2), X (α-1) and XXIV (α-1) as constituents of this fibrous biomaterial (Table 4) [7, 24, 25, 33]. Additionally, peptides derived from collagens type I (α-1 and α-2), II (α-1), III (α-1), V (α -2), VI (α-1 and α-2), X (α-1), XVII (α-1) and XVIII (α-1) were detected in the matrix of chicken eggshell [6, 34, 35]. In summary, a significant structural fibrillar protein, *COL10A1*, was found to be the most highly up-regulated gene in the white isthmus, while a number of other collagen genes are also over-expressed in this oviductal segment.

In view of our demonstration that a number of collagens were up-regulated in the white isthmus, we searched for over-expression of genes encoding collagen processing enzymes. Lysyl oxidase is a copper-sensitive enzyme that is associated with the formation of collagen cross-links. Its activity is located in the copper-rich region of the white isthmus of the hen oviduct [36], and can be detected in the eggshell membranes where it participates in the cross-linking of eggshell membrane proteins [37]. A deficiency in hen dietary copper elicited a disruption in eggshell membrane formation [15]. In chicken, there are 5 lysyl oxidase genes (lysyl oxidase: *LOX*, and lysyl oxidase homologs 1 to 4: *LOXL1, LOXL2, LOXL3, LOXL4*); the Del Mar 14 K Chicken Integrated Systems microarray contains cDNAs for detection of *LOX* and *LOXL1, LOXL2* and *LOXL3*.

Our results show that *LOXL3* was over-expressed 1.27-fold in the WI/Ma group, but was not over-expressed in the WI/Ut comparison (Table 4). *LOXL3* was previously identified as a constituent of eggshell membranes [17, 18, 31] and the shell matrix by proteomics analyses [38]. We also identified *LOXL2* as highly over-expressed, especially in the WI/Ut contrast (18.64-fold) (Table 4). Lysyl oxidase homolog 2 was one of the most abundant proteins present at the earliest stage of shell calcification, suggesting a predominant role amongst the lysyl oxidases [6]. Although not over-expressed, *LOXL1* was expressed at extremely high (saturating) levels in all three tissues (not shown). Our results indicate that *LOXL1*, *LOXL2* and *LOXL3* are likely to participate in the cross-linking of collagen and other fibrillar proteins during formation of the eggshell membranes. While *LOXL4* was not identified in this study, recent proteomics analysis confirms its presence in the eggshell membranes [19].

Prolyl 4-hydroxylase subunit α-2 is a collagen-associated protein encoded by the *P4HA2* gene that is over-expressed in the white isthmus (1.21-fold and 1.64-fold in the WI/Ut and WI/Ma contrasts, respectively). It is a component of prolyl 4-hydroxylase, a key enzyme required for collagen synthesis which catalyzes the formation of 4-hydroxyproline that is essential to the proper three-dimensional folding of newly synthesized procollagen chains [39, 40]. It is noteworthy that prolyl 4-hydroxylase beta polypeptide (*P4HB*) was also identified as over-expressed in the WI/Ma contrast. Peptidyl-prolylcis-trans isomerase C (*PPIC*) is also a collagen-associated protein which catalyzes the cis-trans isomerization of proline imidic peptide bonds. For example, *in vitro* refolding of denatured type III collagen is rate-limited by cis-trans isomerization of the peptide bond by peptidyl-prolyl cis-trans isomerase [41]. *PPIC* was found to be over-expressed 1.28 and 1.51-fold in the WI/Ut and WI/Ma contrasts, respectively (Table 4) while proteomics analyses confirm its presence in the shell membranes [17] as well as in the shell matrix during the initial phase of calcification [6]. Therefore, genes encoding collagens as well as collagen-processing enzymes, which are probably involved in the synthesis of additional fibrillar proteins, are upregulated in the white isthmus.

### Fibrillin

A structural constituent identified by our analysis for the first time as a potential extracellular component of the eggshell membrane fibres is *FBN1*. Its over-expression level was 45.89-fold in the WI/Ut contrast and 2.53-fold in the WI/Ma contrast (Table 3); however, fibrillin-1 has only been identified in uterine fluid bathing weak eggs [38] and has yet to be identified in proteomics studies of the shell membranes and shell matrix. This suggests that fibrillin is highly specific to the white isthmus region of the oviduct and possibly is a highly insoluble constituent of the shell membrane fibers. Fibrillin is the major constitutive element of extracellular microfibrils and has widespread distribution in both elastic and non-elastic connective tissues throughout the body [42]. The molecular weight of chicken fibrillin-1 is predicted to be about 335 kDa, with 12 % cysteine residues; similar to human fibrillin (350 kDa; 14 % cysteine, of which one-third appears to be in the free reactive sulfhydryl form) [43]. About 75 % of fibrillin is composed of 46 EGF-like repeats, which are cysteine-rich domains originally found in human epidermal growth factor. Forty-three of these repeats satisfy the consensus for calcium binding, which is a known property of fibrillin [44]. Thus, fibrillin-1 is a likely candidate as a structural protein in the eggshell membranes forming microfibrils that contribute to the elasticity of eggshell membranes.

### The cysteine-rich eggshell membrane protein (CREMP)

Another over-expressed gene, *CREMP* (EST accession BM439825.1; XP_001236415), was strongly expressed in a white isthmus– specific manner (58.08-fold in WI/Ma; 70.03-fold in WI/Ut) (Table 3). The corresponding protein fragment was recently identified in chicken eggshell membranes as potentially significant cysteine-containing constituent [17, 18]. This EST coding for a disulfide-rich sequence was originally annotated as similar to spore coat protein SP75, since a related gene was first identified in slime mold [45]. When calculated on a percent amino acid composition basis, fibrillin-1 (XP_413815.4) and CREMP (BM439825.1) are 12.8 and 13.8 % cysteine, respectively, in contrast to collagen X (α-1) (P08125) which is only 0.2 % cysteine. Thus, fibrillin-1 and CREMP are both in agreement with the literature values for whole eggshell membrane (i.e., 10.1 ± 0.7 % cysteine), as summarized by Kodali et al*.* [18]. These observations demonstrate that either (or both) CREMP and fibrillin-1 proteins could account for the relatively high cysteine content of eggshell membranes, in contrast to collagen X.

A number of genes encoding enzymes associated with disulfide mediated protein cross-linking were over-expressed in the white isthmus. Thioredoxin is encoded by *TXN* (Table 3) and participates in many biological processes as an antioxidant by facilitating the reduction of other proteins by cysteine thiol-disulfide exchange [46]; also, protein disulfide isomerase (*PDIA5*) catalyzes the formation of disulfide bonds (Table S1). Kodali et al. [18] showed that a reduced recombinant cysteine-rich eggshell membrane protein is an efficient thiol substrate of sulfhydryl oxidase (*QSOX1*) which is detected in the vitelline membrane, egg white, eggshell and hen oviduct tissue [18, 34, 35, 47–52]. Sulfhydryl oxidase 1 is a secreted disulfide catalyst recently shown to control extracellular matrix composition and function [53]. Close inspection of the individual fluorescence intensity data for the 3 cDNA clones annotated as *QSOX1* revealed a high and equivalent expression in all three segments of the oviduct (Ma, WI and Ut; data not shown). Recent proteomics studies have confirmed the presence of sulfhydryl oxidase 1 in eggshell membranes, mammillary cones and uterine fluid during the initial phase of eggshell calcification [6, 7, 33]. Therefore, sulfhydryl oxidase is likely to participate in eggshell membrane formation by catalyzing the cross-linking of membrane fibres.

Several other genes are highly expressed in the white isthmus (Table 3). *FCGBP* interacts with the Fc portion of IgG and with mucin 2 (*MUC2)* [54]. MAX interactor 1 (*MXI1*) belongs to the family of proteins that function as potent antagonists of MYC oncoproteins. The antagonism relates to their ability to compete with MYC for the MAX protein and for consensus DNA binding sites [55]. Another highly over-expressed gene was chicken R3H domain and coiled containing 1 (*R3HCC1*); the function of the protein encoded by this conserved gene is unknown although it is predicted to bind ssDNA or ssRNA in a sequence-specific manner (Entrez Gene annotation). LIM and calponin homology 1 (*LIMCH1*) is also highly over-expressed in the white isthmus; it contains two domains: the calponin homology domain is actin-binding and the LIM domain is a small protein-protein interaction domain with two zinc fingers [56]. The D-3-phosphoglycerate dehydrogenase (*PHGDH*) enzyme is involved in the early steps of L-serine synthesis in animal cells [57]. Sorbitol dehydrogenase (*SORD*) is an enzyme of glucose metabolism which catalyzes the interconversion of polyols and their corresponding ketoses, and produces fructose [58]. *HPX* encodes a plasma glycoprotein that binds heme with high affinity. The encoded protein transports heme from the plasma to the liver and could be involved in protecting cells from oxidative stress [59]. It is noteworthy that several proteomics analyses have found hemopexin to be associated with the eggshell membranes, mammillary cones and uterine fluid during the early stages of shell mineralization [6, 7, 17, 18, 33]. Tudor domain containing 9 (*TDRD9*) is a probable ATP-binding RNA helicase which plays a central role during spermatogenesis by repressing transposable elements and preventing their mobilization, which is essential for germline integrity [60]. Adaptins (*AP1G1*) are important components of clathrin-coated vesicles transporting ligand-receptor complexes from the plasma membrane or trans-Golgi network to lysosomes [61]. *TMEM63C* is an uncharacterized gene which is conserved from zebrafish to human (Entrez Gene annotation).

### Proteoglycans

Proteoglycans with keratan sulfate and dermatan sulfate epitopes have been implicated in the assembly and function of eggshell membranes and shell matrix, respectively [29]. Ovocleidin-116, expressed in uterine cells and found to be most abundant during the rapid growth phase of mineralization, is the core protein of ovoglycan, a variably modified dermatan sulfate proteoglycan detected in SDS-PAGE at 120 and 220 kDa [6, 62]. Previous studies have reported that a keratan sulfate epitope is located at the sites of calcium carbonate nucleation on the eggshell membranes during the initiation of calcification [27]. Secretion of this keratan sulfate epitope (termed “mammillan”) coincides with the formation of the mammillary cones [28, 29]. However, the core protein of this putative keratan sulfate proteoglycan has not yet been cloned or characterized. Therefore, we examined the list of over-expressed genes in the white isthmus carefully to detect possible proteoglycans, proteoglycan-processing enzymes or keratan sulfate annotations. The Del-Mar 14 K Chicken Integrated Systems Microarray contained 23 unique cDNA clones annotated as proteoglycans, none of which was over-expressed in either WI/Ut or WI/Ma contrasts. One over-expressed gene, *ADAMTS1* (Table S1), encodes a member of the ADAMTS (a disintegrin and metalloproteinase with thrombospondin motif) protein family, with proteoglycan-degrading activity that has a role in the remodeling of extracellular matrix and is involved in mineralized nodule and bone formation [63]; ADAMTS-1 is also capable of binding to sulfated glycosaminoglycan chains [64].

Keratan sulfate is composed of repeating disaccharide units of Galb1-4GlcNAc (poly-N-acetyllactosamine) with sulfate groups at the C6 position of the Gal and GlcNAc residues [65]. The sulfation of oligosaccharides is carried out in the lumen of the Golgi apparatus by the transfer of a sulfate group from 3’-phosphoadenosine 5’-phosphosulfate (PAPS) to a precursor oligosaccharide. *SULT1B1* (sulfotransferase family, cytosolic, 1B, member 1), which utilizes PAPS as sulfonate donor to catalyze the sulfate conjugation of many hormones, neurotransmitters, drugs and xenobiotic compounds, is expressed at very high (even saturating) levels in all three oviduct tissues (not shown). *GNS*, which codes for N-acetylglucosamine-6-sulfatase precursor, a calcium-dependent lysosomal enzyme associated with keratan breakdown, is specifically upregulated in the white isthmus (1.28-fold and 1.29-fold in the WI/Ut and WI/Ma contrasts, respectively).

### Calcium-binding proteins

A number of over-expressed genes are related to cellular calcium handling or could mediate Ca^2+^ signaling in various cellular activities (Table 2). The *RCN2* gene encodes reticulocalbin-2, a calcium-binding protein located in the lumen of the endoplasmic reticulum. *RCN2* codes for a protein that contains six conserved regions with similarity to a high affinity Ca^2+^ -binding motif, the EF-hand [66]. *CHP1* is a calcium-binding protein involved in different processes such as regulation of vesicular trafficking, plasma membrane Na^+^/H^+^ exchanger and gene transcription; it is required for the targeting and fusion of transcytotic vesicles [67]. *TMBIM4* is an anti-apoptotic protein which can inhibit apoptosis induced by intrinsic and extrinsic apoptotic stimuli, and modulates both capacitative Ca^2+^ entry and inositol 1,4,5-trisphosphate (IP_3_)-mediated Ca^2+^ release [68].

### Antimicrobial protection

One over-expressed gene in the white isthmus encodes an antimicrobial protein that could contribute to egg innate defense mechanisms. The antimicrobial peptide, gallinacin-10 [also called avian beta-defensin 10 (AvBD-10)] is encoded by the *GAL10* gene [69]. Beta-defensins are a group of cysteine-rich antimicrobial peptides that are effective against Gram-positive and Gram-negative bacteria as well as fungi [70]. Proteomics analyses of egg compartments have detected avian beta-defensins in multiple egg components [35, 49]. This study reports a marginal but significant over-exprresion of *GAL10* with a 1.17-fold in the WI/Ut contrast and a 1.15-fold increase in the WI/Ma contrast (Additional file 1). A recent proteomics study identified AvBD-10 associated with eggshell membranes from both fertilized and unfertilized eggs [7]. Bioinformatics indicate that AvBD-10 possesses a signal peptide and is categorized as “defense response to bacterium” by the biological process GO term analysis. Transcriptomic, proteomics and bioinformatics evidence all suggest that AvBD-10 (*GAL10*) is an antimicrobial defense molecule enriched in the white isthmus region of the oviduct.

## Conclusions

Gene expression profiling of the chicken oviduct during the formation of eggshell membranes has revealed numerous over-expressed genes; bioinformatics tools were applied to analyze these genes as well as their encoded proteins. This method allowed us to identify 135 over-expressed transcripts in the white isthmus, corresponding to as many as 107 annotated genes, which we propose to be involved specifically in the formation of the eggshell membrane fibres. Our study provides strong support for the role of collagen X and CREMP in eggshell membrane structure, and revealed that fibrillin-1 is another component specific to the fabrication of the eggshell biomaterial.

The detailed annotation of these genes and their encoded proteins, leading to a clear knowledge of their functional properties, will be an important step towards determining their role in eggshell membrane formation. The chicken eggshell membranes are natural biomaterials that have potential uses in clinical, industrial, cosmetic and various other industries [16]. Because of many potential uses for eggshell membranes, identifying the protein components is the first step to develop and improve the utilization of the eggshell membrane in various fields. However, it has been difficult to analyze the protein components of the eggshell membrane due to its insolubility and highly cross-linked nature.

Eggshell membrane fibres are extremely stable and function as a natural filter to defend the egg against bacteria. We have identified a potential antimicrobial protein component of the membranes and clearly defined abundant structural elements which may have potential applications. This information could lead to novel strategies to optimize egg antibacterial defenses to reduce the risks of food-borne disease.

## Methods

### Ethics statement

All experiments, including all animal-handling protocols, were carried out in accordance with the European Communities Council Directives of 24 November 1986 (86/609/EEC) concerning the practice for the care and Use of Animals for Scientific purposes and the French ministerial decree 87848 of 19 October 1987 (revised on 31 May 2001) on Animal experimentation under the supervision of authorized scientists (authorization # 7323, delivered by the DDPP, direction départementale de la protection des populations, d’Indre et Loire). The experimental unit UE-PEAT 1295 where the birds were kept has the agreement for rearing birds and for the euthanasia of experimental animals (decree N° B37-175-1 of August 28th 2012 delivered by the Préfecture d’Indre et Loire following the inspection of the Department Direction of Veterinary Services). The protocol was approved by an ethics committee (comité d’éthique de val de Loire, officially registered under number 19 of the French national ethics committee for animal experimentation), under agreement number 00159.02.

### Animal handing and tissue collection

Forty-week old brown egg-laying hens (ISA-Hendrix, brown strain) were caged individually, with a light/dark cycle of 14 h light and 10 h darkness (14 L:10D), with controlled humidity and temperature. The chickens were fed a layer mash recommended by the Institut National de la Recherche Agronomique (INRA) and bird cages were equipped with automatic recording devices that monitor the time of oviposition. One sample from each segment (magnum, white isthmus and uterus) of the hen oviduct was collected from eight hens during the phase of eggshell calcification. The “white isthmus” corresponds to the upper part of the isthmus where the eggshell membrane fibres are secreted. This region can be easily distinguished from the magnum due to a translucent ring indicating a change in cell type [71]. The lower part of the isthmus, also known as the “red isthmus”, is very similar to the uterine tissue. Samples were immediately frozen in liquid nitrogen and stored at −80 °C until RNA isolation.

### RNA isolation and microarray hybridization

The Del-Mar 14 K Chicken Integrated Systems Microarray (NCBI GEO Platform # GPL1731) was used to analyze gene expression in different parts of the hen oviduct during the formation of the eggshell membranes. This microarray system contains total of 14,053 unique cDNAs with 7,937 unique cDNA clones from the neuroendocrine and reproductive systems as well as 9,833 unique cDNA clones from the metabolic and somatic systems [24].

RNA extraction, quality controls, cDNAs labeling and hybridization were performed as previously reported [21]. Briefly, RNeasy Mini Kit (Qiagen, Courtabeouf, France) was used to extract RNA from frozen tissue samples; the samples were also treated with DNase 1 (Macherey-Nagel EURL, France). RNA concentration was determined at 260 nm and its integrity was analyzed using a 1 % agarose gel with an Agilent 2100 Bioanalyzer (Agilent Technologies, Massy, France). RNA samples with a 28S/18S ratio >1.3 were used for labeling and hybridization. The Superscript Plus Indirect cDNA labeling System (Invitrogen, Cergy Pontoise, France) was used to label total RNA (20 μg). Labeled cDNA was synthesized and purified prior to being assessed with a Nanodrop ND 1000 (Nanodrop, Nyxor Biotech, Palaiseau, France).

Hybridization was carried out using the balanced block design; half of the samples were labeled with Alexa Fluor® 555 and the other half were labeled with Alexa Fluor® 647 dyes (Fisher Scientific BioBLock, Illkirch, France). There were two comparisons: white isthmus vs. uterus (WI/Ut) and white isthmus vs. magnum (WI/Ma), where each comparison utilized 8 microarray slides for hybridization with 16 samples (1 sample from each segment for 8 hens. cDNA probes were used for hybridization only if they had an incorporation efficiency >11.4 dye molecules/1000 bases. Prehybridization was performed with 100 μl DIG Easy Hyb buffer (Roche, Indianapolis, IN) for all microarray slides in a humidified chamber for 1 h at 42 °C. The slides were washed with distilled water for 10 min at room temperature. Equal quantities of Alexa Fluor® 555- and Alexa Fluor® 647- labelled cDNA probes from two samples were combined, added to the hybridization solution, and then denatured at 100 °C for 2 min. The combined solution was placed on the slides, which were then cover slipped and placed in the hybridization chamber for 16 h at 42 °C. The slides were washed with 0.2X saline sodium citrate (SSC) buffer and sodium dodecyl sulfate (SDS) buffer for 15 min at 42 °C, and then washed with 0.2X SSC for 15 min at room temperature. Finally, the slides were washed with distilled water and dried by low speed centrifugation. A GenePix 4000 B scanner (Axon Molecular Devices, California, USA) was used to scan the microarray slides, Alexa Fluor® 555 was scanned at 532 nm and Alexa Fluor® 647 was read at 635 nm. GenePix Pro 6.0 software was used to analyze the spot intensity of microarray data. GenePix report (GPR) files containing spot intensity raw data were stored in the BioArray Software Environment (BASE) of SIGENAE (Système d’Information du projet d’Analyse des Génomes des Animaux d’Elevages) and the microarray data was uploaded in the NCBI Gene Expression Omnibus (GEO) database under the series accession numbers GSE17267 and GSE52491.

### Validation of gene expression by qRT-PCR

Sixteen genes, chosen to represent a wide range of gene expression, were selected for verification of transcript abundance using quantitative real time PCR (qRT-PCR), as documented in our previous analysis of this data for uterus-specific over-expression [21].

### Statistical data analysis

Gene expression was compared between the white isthmus and the uterus as well as between the white isthmus and the magnum (2 samples/microarray slide, 1 microarray slide per hen, for a total of eight hens for each comparison). The ‘anapuce’ package in R was used to identify differentially expressed genes [72]. Spot intensities were calculated using the median value. These values were transformed to log2 values and normalized using global locally-weighted regression (Lowess) in order to eliminate any bias arising from the efficiency of fluorescent dye incorporation. Subtraction of the median value corrected a block effect and the spot intensities were kept only when present in >50 % of samples. Gene variance was estimated using a mixture model integrated into the VarMixt method [73] followed by a unilateral statistical *t*-test to identify genes over-expressed in the white isthmus compared to either the magnum or uterus. The Benjamini-Hochberg multiple testing procedure [74] was used to adjust the *P*-values to control the false discovery rate (FDR < 0.05).

### Functional annotation

The clone sequences of the 135 over-expressed transcripts were available from the University of Delaware Chick EST and NCBI databases [75 ]. BlastN analysis of the transcript sequences was performed against the NCBI *Gallus gallus* Refseq RNA and nr nucleotide databases. BlastP analysis of identified proteins was performed against the NCBI non-redundant *Gallus gallus* protein databases.

### Gene ontology enrichment analysis

Gene Ontology (GO) terms represent the functions of proteins encoded by over-expressed genes that are revealed by microarray analysis. Expression Analysis Systematic Explorer (EASE) software from the Database for Annotation, Visualization and Integrated Discovery (DAVID) Bioinformatics Resources 6.7 was used for functional annotation clustering to identify biological themes for 135 over-represented genes in the white isthmus where the formation of the eggshell membranes occurs [76]. Each GO term corresponded to an EASE score (a modified Fisher Exact *P*-Value and high enrichment value), using GOTERM_BP_FAT and GOTERM_MF_FAT. Only GO terms with an EASE score ≤ 0.05 were considered to be significantly enriched.

### Secreted protein identification

Two protein informatic softwares, SignalP 4.1 [77, 78] and SecretomeP 2.0 [79, 80], were applied to identify the over-expressed genes in the white isthmus that encoded sequences specifying either endoplasmic reticulum – mediated export (SignalP) or protein sequences predictive of unconventional secretion (SecretomeP). For SignalP 4.1 analysis, the proteins were only accepted for secretion if the D-score was above the 0.450 cutoff threshold. However, for the SecretomeP analysis the NN-score cutoff was 0.5 for unconventional secretion.

## Additional file

Additional file 1:
**Excel file (.xlsx) containing list of over-expressed transcripts (107 genes) in WI/Ma and WI/Ut contrasts.** (XLSX 26 kb)

## Abbreviations

AvBD: Avian Beta-Defensin
BASE: BioArray Software Environment
CREMP: Cysteine-Rich Eggshell Membrane Protein
DAVID: Database for Annotation, Visualization and Integrated Discovery
EASE: Expression Analysis Systematic Explorer
EST: Expressed Sequence Tag
GEO: Gene Expression Omnibus
GO term: Gene Ontology term
GPR: GenePix Report
KS: Keratan Sulfate
Ma: Magnum
NCBI: National Center for Biotechnology Information
PAPS: 3'-PhosphoAdenosine-5'-PhosphoSulfate
qRT-PCR: quantitative Reverse Transcription-Polymerase Chain Reaction
SDS: Sodium Dodecyl Sulfate
SIGENAE: Système d’Information du projet d’Analyse des Genomes des Animaux d’Élevage
SSC: Saline Sodium Citrate
Ut: Uterus
WI: White Isthmus
